# Provision of carbon skeleton for lipid synthesis from the breakdown of intracellular protein and soluble sugar in *Phaeodactylum tricornutum* under high CO_2_

**DOI:** 10.1186/s12896-019-0544-4

**Published:** 2019-07-26

**Authors:** Aiyou Huang, Songcui Wu, Wenhui Gu, Yuanxiang Li, Xiujun Xie, Guangce Wang

**Affiliations:** 1 Key Laboratory of Experimental Marine Biology, Institute of Oceanology, Chinese Academy of Sciences, Qingdao, 266071 China; 2 Laboratory for Marine Biology and Biotechnology, Qingdao National Laboratory for Marine Science and Technology, Qingdao, 266071 China; 3 Center for Ocean Mega-Science, Chinese Academy of Sciences, 7 Nanhai Road, Qingdao, 266071 China

**Keywords:** *Phaeodactylum tricornutum*, Lipid, High CO_2_ concentration, Origin of carbon skeleton

## Abstract

**Background:**

Increasing CO_2_ emissions have resulted in ocean acidification, affecting marine plant photosynthesis and changing the nutrient composition of marine ecosystems. The physiological and biochemical processes of marine phytoplankton in response to ocean acidification have been reported, but have been mainly focused on growth and photosynthetic physiology. To acquire a thorough knowledge of the molecular regulation mechanisms, model species with clear genetic background should be selected for systematic study. *Phaeodactylum tricornutum* is a pennate diatom with the characteristics of small genome size, short generation cycle, and easy to transform. Furthermore, the genome of *P. tricornutum* has been completely sequenced.

**Results and discussion:**

In this study, *P. tricornutum* was cultured at high and normal CO_2_ concentrations. Cell composition changes during culture time were investigated. The ^13^C isotope tracing technique was used to determine fractional labeling enrichments for the main cellular components. The results suggested that when lipid content increased significantly under high CO_2_ conditions, total protein and soluble sugar contents decreased. The ^13^C labeling experiment indicated that the C skeleton needed for fatty acid C chain elongation in lipid synthesis under high CO_2_ conditions is not mainly derived from NaHCO_3_ (carbon fixed by photosynthesis).

**Conclusion:**

This study indicated that breakdown of intracellular protein and soluble sugar provide C skeleton for lipid synthesis under high CO_2_ concentration.

## Background

Increased CO_2_ emissions have caused ocean acidification, which has greatly affected the photosynthesis process of marine plants. Ocean acidification has not only increased photosynthetic and respiration rates [1], and down regulated the carbon concentration mechanism [2], but also influenced nutrition uptake, and altered the C/N ratio and cell composition in marine phytoplankton [3, 4]. Since marine phytoplankton are the main primary producers in the ocean, changes in their biomass and cell composition will influence the nutritional status of marine zooplankton. Therefore, it is of great importance to study phytoplankton growth and cell composition changes under high CO_2_ conditions. For systematic research, it is important to select model species with a clear genetic background, that are easy to transform, and perform in metabolic flow analysis.

As the main components of marine phytoplankton, marine diatoms produce approximately 20% of global primary productivity and play an important role in inorganic C fixation and material cycles [5, 6]. *Phaeodactylum tricornutum* is a pennate diatom with the characteristics of small genome size, short generation cycle, and easy to transform [7]. Since its whole genome sequencing has been completed [8], it has clear genetic background and is one of the most promising candidates for studying photosynthetic physiology in single-cell algae. *P. tricornutum* is rich in polyunsaturated fatty acids and can therefore be used as food for aquaculture animals [9]. It has the potential to be used as raw biodiesel materials as it can accumulate oil under conditions such as nitrogen deficiency [10]. Its main pigment is fucoxanthin, which is reported to have antioxidant, cancer prevention, free radical scavenging, and weight loss effects [11]. Therefore, the study of the C flow distribution mechanism in *P. tricornutum* under high CO_2_ has not only fundamental research significance but also important application value.

The physiological and biochemical responses of *P. tricornutum* under high CO_2_ have been reported. Wu et al. (2010) found that under short term exposure to high CO_2_ concentration, the growth, photosynthetic, and respiration rates of *P. tricornutum* increased, while carbon concentrating mechanism activity decreased [12]. After long-term adaptation, growth and respiration rate have been shown to decrease [13]. Li et al. (2012) found that high CO_2_ had no effect on the growth of *P. tricornutum* [14]. These inconsistent results might be caused by different light intensities.Besides, stress such as C-limitation might also trigger modifications of the carbon flow in *P. triconutom* [15, 16]. These results suggest that factors such as culture time, light intensity, and nutritional status can affect the response of *P. tricornutum* to high CO_2_, which are important for revealing the physiological and ecological processes of marine diatoms under high CO_2_. However, since this research is limited to growth and photosynthetic physiology only, the molecular mechanism of the response process is not fully understood. Based on genomic data, Levering et al. (2017) integrated the metabolic network and regulation mode of *P. tricornutum*, and predicted its global regulation mechanism under different CO_2_ concentrations (400 ppm and 5,000 ppm) at the transcriptome level [17]. To verify these predictions, a large number of experiments should be conducted.

In a previous study, the current authors found that the growth rate, lipid content, and fucoxanthin content of *P. tricornutum* were significantly increased in short-term culture under high CO_2_ (2,000 ppm). The key enzymes of the Calvin cycle and pentose phosphate pathway were elevated at both transcriptional and enzymatic levels [18]. This suggested that *P. tricornutum* readjusts central C metabolism under high CO_2_, and supplements its growth and lipid synthesis by high-speed operation of pentose phosphate pathway. These results provide theoretical and technical support for the study the response of *P. tricornutum* to high CO_2_. However, the molecular mechanism of its response process has not been comprehensively analyzed, and further research is needed.

In this study, *P. tricornutum* was cultured at different CO_2_ concentrations. Changes in cell composition with culture time were determined. The ^13^C isotope tracing technique was used to detect the origin of C skeletons. This study reveals the C flow distribution mechanism of *P. tricornutum* in response to high CO_2_ concentration.

## Results

### Influence of CO_2_ concentration on growth

**Fig. 1:**
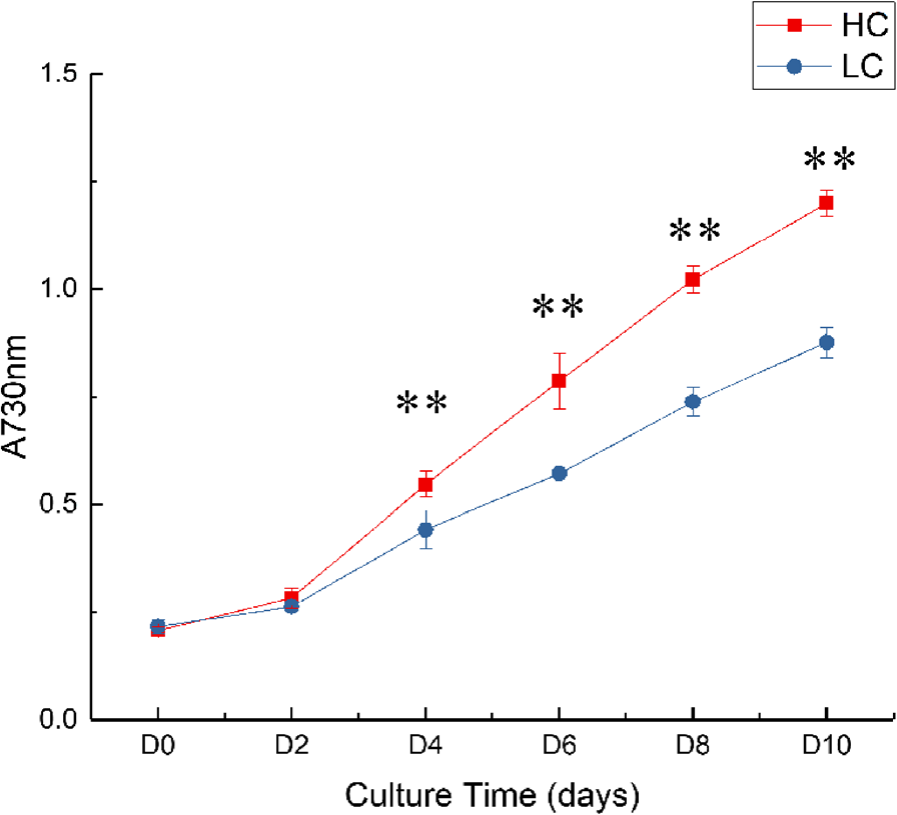
Growth of *P. tricornutum* under HC and NC conditions. *Y* axis, *A*_730nm_; *X* axis, culture time (days); HC, High CO_2_ condition (~ 2,000 ppm CO_2_); NC, normal CO_2_ condition (~ 400 ppm CO_2_). Error bars represent the standard deviation of three replicates

The growth rate of *P. tricornutum* showed significant differences under ~ 400 ppm of CO_2_ (NC) or high CO_2_ of ~ 2,000 ppm (HC) (Fig. 1). Under HC, A_730nm_ was ~ 0.20 at the beginning of inoculation, ~ 0.80 on D6, and ~ 1.21 on D10. Under NC, A_730nm_ was ~ 0.20 at the beginning of inoculation, ~ 0.57 on D6, and ~ 0.87 on D10. These results were consistent with a previous study [18].

### Changes in cell composition with culture time under HC and NC

#### Total lipid content

**Fig. 2:**
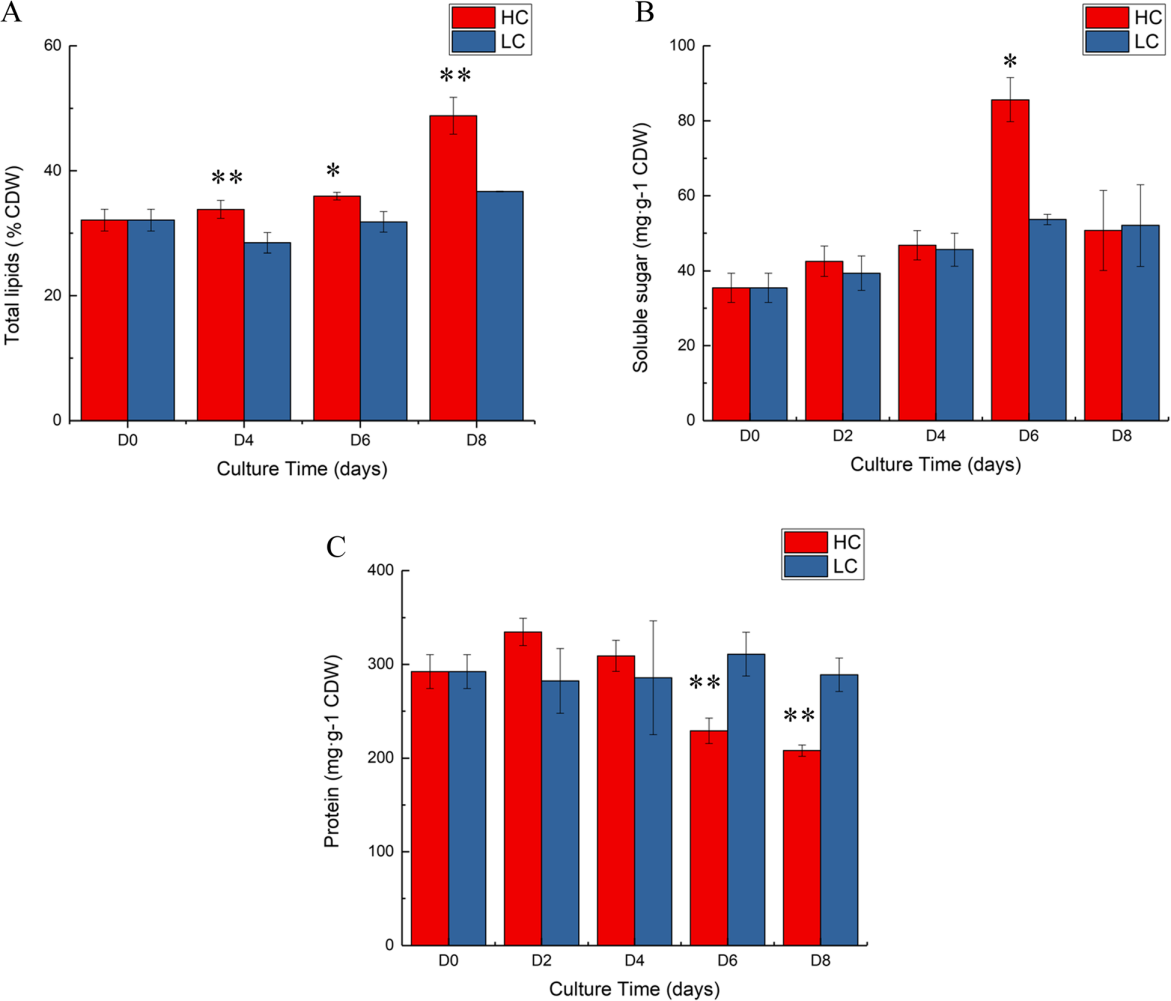
Changes of major cell compositions with culture time under HC and NC conditions. **a**. Changes in total lipid content. **b**. Changes in total soluble sugar. **c**. Changes in total protein content. *Y* axis, % cell dried weight (%CDW) for total lipid and mg·g^− 1^ for sugar and protein; *X* axis, culture time (days). Error bars represent the standard deviation of three replicates

Total lipid content changed relatively little at the beginning of cultivation (within 6 days after inoculation) under both HC and NC. After 8 days of cultivation, total lipid content increased a little under NC, but was significantly enhanced under HC (Fig. 2a). This was consistent with previous findings [18]. During the entire cultivation, total lipid content was higher under HC than under NC at the same time point.

#### Soluble sugar content

Under NC, soluble sugar content increased gradually within 8 days after inoculation. While under HC, soluble sugar content increased sharply within 6 days after inoculation, then decreased significantly on D8. There was little difference between soluble sugar content under HC and NC during the entire cultivation, except on D6, when soluble sugar content under HC was significantly higher than that under NC (Fig. 2b).

#### Total protein content

Under NC, total protein content changed little within 8 days after inoculation. While under HC, total protein content increased a little on D4, then decreased gradually on D6. On D8, total protein content decreased significantly under HC. On D2, total protein content under HC was higher than that under NC. While on D6 and D8, it was significantly lower than that under NC (Fig. 2c).

### Fractional labeling s of major cellular components under HC and NC

To determine the main C flow of photosynthesis and to track the origin (de novo synthesis through photosynthesis or degradation of intracellular substances) of the C skeleton of the major cellular components, the ^13^C isotope tracing technique was used and the (fractional labeling) FLs of major cellular components under HC and NC were analyzed. In general, for the same substance, the FL was higher under NC than under HC.

#### Fractional labeling of lipids

**Fig. 3:**
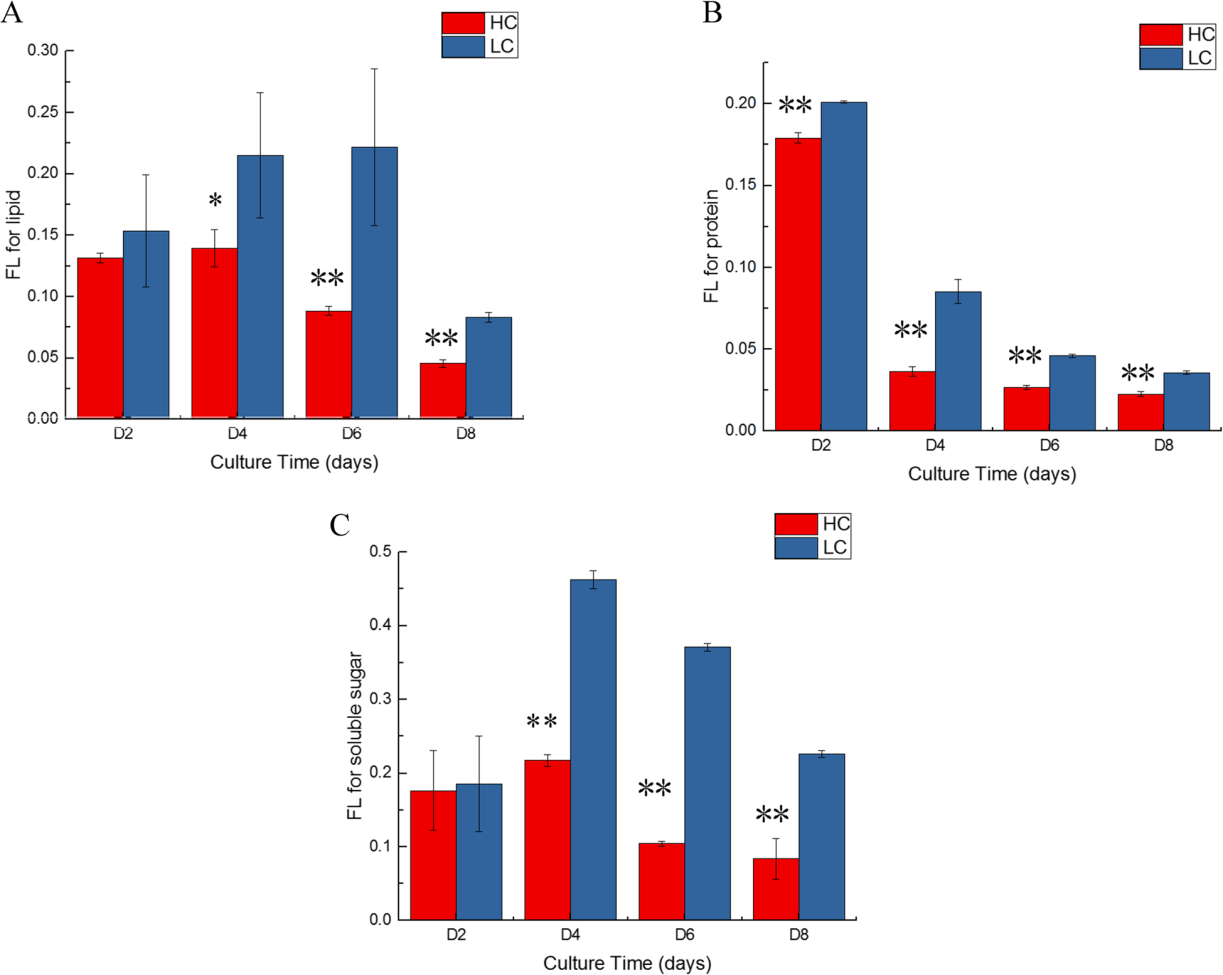
Changes in ^13^C fractional labeling (FLs) enrichment with culture time for major cellular components under HC and NC conditions. **a**. FLs of total lipid. **b**. FLs of total soluble sugar. **c**. FLs of total protein. *Y* axis, FL; *X* axis, culture time (days). Error bars represent the standard deviation of three replicates

To analysis the FLs of total lipids, total lipids were derivatized to butyl amides and the FLs of fatty acids were measured. Based on GC-MS, five fatty acids were detected. Among these, stearic acid was detected throughout all samples, and its abundance was much higher than that of the other fatty acids. Therefore, its FL was used to represent the FLs of lipids. Cells on D1, D3, D5, and D7 were labelled and sampled on D2, D4, D6, and D8 (1 day after labelling), respectively. Under HC, the FL of stearic acid increased a little on D4. After that, the FL decreased with increasing culture time. The FL on D8 (~0.045) was significantly lower than that on D2 (~ 0.131). Under NC, the FL increased on D4 and D6. On D8 The FL decreased to ~ 0.083 (Fig. 3a).

#### Fractional labeling of soluble sugar

To analysis the FL of soluble sugar, soluble sugar was converted into monosaccharides by hydrolysis. Monosaccharide was then derivatized by Bis(trimethylsilyl) trifluoroacetamide. The FL of glucose was measured and used to represent the FL of soluble sugar. Under HC, the FL was ~ 0.176 on D2, increased significantly on D4 (~ 0.217), then decreased rapidly on D6 (0.103). On D8, FL was ~ 0.083. The changing trend of FL under NC was similar to that under HC, except that the level was higher under NC at the same time point. On D8, the FL was still ~ 0.229 under NC (Fig. 3c).

#### Fractional labeling of protein

To analyze the FLs of protein, total protein was converted into amino acids by hydrolysis. Amino acids were then derivatized by N-tert-butyldimethylsilyl-N-methyltrifluoroacetamide. The average FL of all amino acids was used to represent the FL of total protein (Fig. 3b). Under HC, the FL was high only on D2 (~ 0.179), then it decreased significantly on D4 (~ 0.036). On D8, the FL was very low (~ 0.022). Under NC, the FL was high on D2 (~ 0.201), then decreased on D4 (~ 0.085) and D6 (~ 0.046). On D8, the FL was very low (~ 0.035).

### Nitrate concentration in the medium

**Fig. 4:**
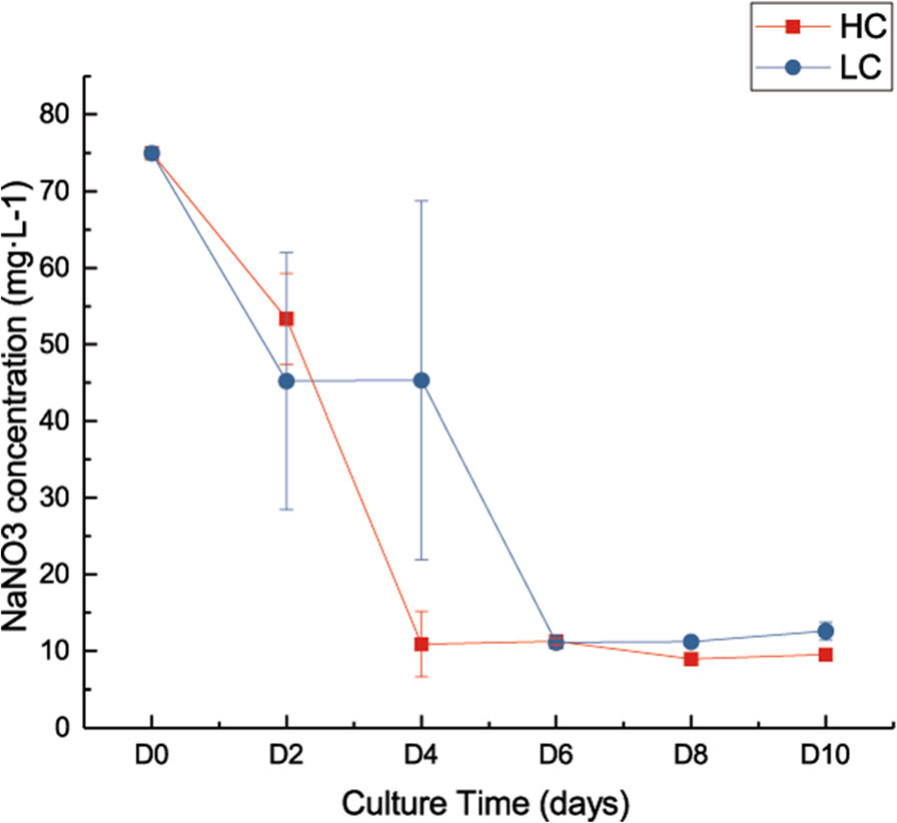
Changes in nitrate concentration in the medium with culture time. *Y* axis, mg·L^− 1^; *X* axis, culture time (days). Error bars represent the standard deviation of three replicates

We measured the concentration of nitrate concentration in the medium. The results showed that nitrate in the HC group and the NC group were exhausted on D4 and D6, respectively (Fig. 4). That is, HC *P. tricornutum* was exposed to nitrogen deficiency earlier than in NC.

## Discussion

### High CO_2_ and nitrogen deficiency might prompt lipid accumulation

With the rapid growth of *P. tricornutum* under HC, abundant nitrogen must be consumed. With the prolongation of culture time, the nitrogen source in the medium would be gradually exhausted. Nitrate was exhausted earlier under HC (Fig. 4). We speculated that nitrogen deficiency stress is the switch that triggers the adjustment of central C metabolism and the start of lipid accumulation in *P. tricornutum* under HC. In our previous study we detected the pH of the growth medium and found that under NC the medium pH increased gradually from 8.83 to 9.41. Under HC the pH decreased on day 1 and then gradually increased to 8.32 [18]. Differences in pH would influence the dissolution of CO_2_. These factors might also impact lipid accumulation under HC and NC.

### *P. tricornutum* provides C skeleton for lipid synthesis by degradation of soluble sugar and protein under HC

The changes in soluble sugar, total lipids, and protein contents were further compared under HC and NC concentrations. Under HC, the contents of total lipids increased on D8, while the contents of soluble sugars and proteins initially increased and then decreased on D8. Under NC, the contents of total lipids and protein changed relatively little, while the contents of soluble sugars increased gradually. These results suggest that under HC *P. tricornutum* might provide C skeleton for lipid synthesis through degradation of soluble sugars and proteins.

### ^13^C labeling experiment provided additional evidence for the origin of C skeleton for fatty acid synthesis under HC

In order to trace the origin of C skeleton for various cell components, *P. tricornutum* was labelled at different time points during cultivation and the labeling degree of soluble sugar, total lipids, and protein was determined. In general, if the cell is labeled at a time point when the synthesis of one substance was vigorous, then the labeling degree of the corresponding substance is high.

Under HC, the protein content increased on D2, then decreased significantly. This indicated that protein synthesis was vigorous at the beginning 2 days of cultivation. Accordingly, the labeling degree of amino acids under HC was the highest on D2 (~ 0.176), then decreased significantly to ~ 0.036 on D4. On D6 and D8, the values were ~ 0.026 and ~ 0.022, respectively, which was similar to the value of unlabeled cells. This indicated that under HC, de novo synthesized amino acids through photosynthetic C fixation decreased after D4. Under NC, protein content changed relatively little. Accordingly, the labeling degree of amino acids was maintained above 0.04 at the beginning 6 days after cultivation. On D8, the value decreased to ~ 0.036. This indicated that on D8, de novo synthesized amino acids through photosynthetic C fixation decreased.

Under HC, the soluble sugar content increased within 6 days after inoculation and then decreased on D8. Accordingly, the labeling degree of sugar was highest on D4 (0.217) and then decreased to 0.104 on D6. This indicated that de novo synthesized sugar through photosynthetic C fixation had decreased on D6. On D8, the value was ~ 0.083, indicating that there was still a small part of sugar was de novo synthesized through photosynthetic C fixation. Under NC, the soluble sugar content increased rapidly initially and then stabilized. Correspondingly, the labeling degree of sugar increased rapidly on D4 (from ~ 0.185 to ~ 0.463), then decreased slowly. On D8, the value was still 0.231, indicating that sugar de novo synthesis remained in progress.

This was not the case for lipids. Under NC, total lipid content did not change much within 6 days of culture. Consistently, the labeling degree did not change much. However, on D8, when total lipid content increased slightly, the labeling degree did not increase but decreased significantly to ~ 0.083. Under HC, total lipid content increased significantly on D8, yet the labeling degree decreased to ~ 0.045. This indicated that the C skeleton needed for fatty acid C chain elongation in lipid synthesis under HC is not mainly derived from NaHCO_3_ (C fixed by photosynthesis), but from the degradation of intracellular substances (such as proteins and soluble sugars).

## Conclusions

This study indicated that the degradation of intracellular substances (such as proteins and soluble sugars) provide the C skeleton needed for fatty acid C chain elongation in lipid synthesis under HC.

## Methods

### Strains and culture conditions

Axenic cultures of *P. tricornutum* (IOCAS-001) were maintained in the laboratory [19]. Cells were cultivated with conditions described previously [19] (20 °C, ~ 100 μmol m^− 2^ s^− 1^ cool white fluorescent light, 12 /12 h light-dark cycle) in artificial seawater (NaHCO_3_ was not contained) [20] with f/2 [21] inorganic nutrients (containing 75 mg L^− 1^ NaNO_3_, 5 mg L^− 1^ NaH_2_PO_4_·2H_2_O, 3.2 mg L^− 1^ FeCl_3_·6H_2_O, 1.8 mg L^− 1^ EDTANa_2_, 20 mg L^− 1^ Na_2_SiO_3_·9H_2_O), trace elements, and vitamins (filter-sterilized). The initial pH was ~ 8.8 and was not controlled in the process of cultivation. Cell growth was monitored as described [18, 19]. For CO_2_ treatment, cultures were cultivated with 1.5-L medium in 2-L flasks, continuously aerated with air (containing ~ 400 ppm CO_2_, NC) or with a mixture gas of air and CO_2_ (containing ~ 2000 ppm CO_2_, HC) at a constant flow rate of ~ 150 ml min^− 1^. There were three replicates for each treatment. For cell composition analysis, cells were harvested at 2 days, 4 days, 6 days, and 8 days after inoculation (D2, D4, D6, and D8, respectively). After 5,000 *g* centrifugation for 4 min at 20 °C, the cell pellet washed with steam-sterilized artificial seawater, and then frozen immediately in liquid nitrogen and stored at − 80 °C.

### ^13^C labeling experiment

Cultures were grown in four replicates in 2-L flasks containing 1.5-L medium. For ^13^C labeling, ^13^C-NaHCO_3_ (Cambridge Isotope Laboratories) was added to a final concentration of 0.174 g l^− 1^ to flasks No.1, No.2, No.3, and No.4 at D1, D3, D5, and D7, respectively. Samples were collected at 1 day after labeling (D2, D4, D6, and D8) as described above.

#### Sample preparation and GC-MS analysis

GC-MS was performed with an Agilent 6890–5973 GC-MS system. Agilent HP-5MS column (30 m × 0.25 mm × 0.25 μm) was equipped and He was the carrier gas.

Analysis of sugar labelling enrichment was conducted as described previously [22]. Briefly, the labelled samples were hydrolyzed with 6 mol l^− 1^ HCl at 110 °C for 3 h and dried at ~ 80 °C overnight, dissolved in 100 μL of anhydrous pyridine, and then derivatized with 50 μL of Bis(trimethylsilyl) trifluoroacetamide. The reaction was conducted at 70 °C for 3 h. After centrifuged at 10,000 *g* for 5 min, the supernatant was collected and filtered with 0.22 μm pore size filters. GC-MS was performed as previously described [22, 23].

For labelling enrichment analysis of protein, samples were hydrolyzed with 6 mol l^− 1^ HCl at 110 °C for 12 h, dried overnight under ~ 80 °C, and dissolved in 100 μL of anhydrous pyridine, and then derivatized with 50 μL of N-tert-butyldimethylsilyl-N-methyltrifluoroacetamide. The reaction was conducted at 85 °C for 60 min and centrifuged and filtered as described above. GC-MS was performed as previously described [19, 23].

For labelling enrichment analysis of lipids, 1 ml hexane was added to ~ 5 mg freeze-dried algal powder, stirred vigorously for 5 min, and centrifugated at 8,000 *g* for 5 min. The supernatant was recovered and the process repeated three times. Hexane aliquots were combined and evaporated at ~ 20 °C. Butyl amide reactions were performed as previously described [24]. 20 μL of standards (triolein, tripalmitin, or tristearin with a concentration of 25 μL/mL) or total lipids extracted from 5 mg cell pellets were dissolved in 3 ml hexane, mixed with 2 ml n-butylamine, and maintained at ~ 70 °C for 48 h. A 1 mL-sample of 4 M HCl was added to quench the reaction. The hexane phase was recovered and analyzed by GC-MS [24].

### GC-MS data processing

The GC-MS data were analyzed as described previously [23, 25–27]. The mass isotopomer distribution vector (MDV) for fragments from glucose, amino acids, and fatty acids were assigned according to formula (1). The natural abundance of stable isotopes of C, N, O, H, Si, and S were corrected and the fractional labeling enrichments (FL) were calculated according to formula (2).

1 $$ MDVa=\left[\begin{array}{c}\left({m}_0\right)\\ {}\left({m}_1\right)\\ {}\vdots \\ {}\left({m}_n\right)\end{array}\right]\;\mathrm{with}\;{\sum}_{i=0}^n{m}_i=1 $$

2 $$ FL=\frac{\sum_{i=0}^ni\cdot {m}_i}{n\cdot {\sum}_{i=0}^n{m}_i} $$

### Total lipid analysi

Total lipids content was determined as described previously [28, 29]. Briefly, 1 ml of methanol/chloroform (1:1) was added to ~ 10 mg freeze-dried cells, stirred vigorously for 5 min, and mixed with 0.3 ml of 0.2 M H_3_PO_4_ (containing 1 M KCl). The mixture was centrifugated for 5 min at 5,000 *g* and the solvent phase was recovered. After three times of extraction, the solvent phases were combined, washed with distilled water for three times, and then evaporated at ~ 20 °C within a fume hood. The total lipid content was weighed and expressed as % cell dry weight (CDW).

### Total soluble sugar analysis

Total soluble sugar content was analyzed as described previously [30, 31]. Briefly, 1 ml of 80% ethanol was added to ~ 5 mg freeze-dried cells, stirred vigorously for 5 min, incubated for 15 min at 68 °C, and then centrifuged for 5 min at 8,000 *g*. The supernatant was collected and merged after three times of extraction, and evaporated at 85 °C to ~ 0.3 ml, made up to a total volume of 1 ml with distilled water and used for the soluble sugar analysis. 0.2 ml sample was mixed with 1 ml of anthrone solution (2 g anthrone in 1 L 72% (v/v) H_2_SO_4_)and incubated at 100 °C for 8 min, and the absorbance under 625 nm was detected. Glucose was used as standard.

### Total protein analysis

Total protein content was determined as previously described [24]. Briefly, ~ 5 mg of freeze-dried algal powder was hydrolyzed in 100 μL of 1 M NaOH and then incubated in a water bath at 80 °C for 10 min. Following this, 900 μL H_2_O was added to the hydrolysate to bring the volume to 1 mL. The mixture was centrifuged for 15 min at 12,000 *g* and the supernatant was recovered. After three times of extraction, all the resulted supernatants were merged, and analyzed using a bicinchoninic acid protein assay kit (cat no. P0010S). Bovine serum albumin was used as the standard.

### Determine of nitrate concentration in the medium

The concentration of nitrate in the medium was determined with a Multi N/C 2100S Analyzer (Analytikjena, Germany) equipped with a solid-state electrochemical detector (ChD). Sodium nitrate was used as the standard. The injection volume was 250 μL and the linear range was 0.2–5 mg L^− 1^. The temperature of the combustion tube was controlled at 800 °C, and the maximum integration time was 200 s.

### Statistical analysis

Each treatment was conducted in 3 replications. Student t-test were used to detect the significance of differences among samples, and the statistical significance level was set at *P*-value < 0.05.

## Data Availability

The datasets used and/or analysed during the current study are available from the corresponding author on reasonable request.

## Abbreviations

CDW: Cell dry weight
FL: Fractional labeling
HC: ~ 2,000 ppm CO_2_
MDV: Mass isotopomer distribution vector
NC: ~ 400 ppm of CO_2_
